# Hypodermic Needles

**Published:** 1898-04

**Authors:** R. E. L. Barnum

**Affiliations:** Richland, Ga.


### HYPODERMIC NEEDLES.

RICHLAND, GA., March 23, 1898.

*Atlanta Medical and Surgical Journal:*

I enclose a little item that may be of interest and some value to many physicians who have hypodermic needles to buy.

When the hypodermic needle becomes plugged up by a particle of rust, or any substance whatsoever, and which cannot be removed, by the little wire that accompanies every syringe, fill the syringe with water, screw on needle, press down upon obstacle by piston-rod, then hold needle at the point of obstruction in the flame of an alcohol lamp, or any lamp as to that matter, and the steam produced thereby will expel the obstructing substance at once.

This does not materially injure the needle, as I have used them for months afterward. It is not necessary to hold the needle in the flame but a moment, and then by expelling water immediately the needle is cooled.

By this simple procedure many an old needle long thrown aside may again be brought into use, and the price of many needles saved in the course of a physician's experience.

Yours truly, R. E. L. BARNUM, M.D.

---

Is sepsis everything? Is traumatism nothing? What causes the death of the woman who perishes on the operating-table or in the obstetric bed twenty-four hours after labor? Does any one mean to say that within twenty-four hours germs have entered that woman's system and killed her? No! It is traumatism which has killed her and this traumatism we must avoid.—*Malcolm McLean.*

